# CLCA2 Interactor EVA1 Is Required for Mammary Epithelial Cell Differentiation

**DOI:** 10.1371/journal.pone.0147489

**Published:** 2016-03-01

**Authors:** Grace Ramena, Yufang Yin, Yang Yu, Vijay Walia, Randolph C. Elble

**Affiliations:** 1 Dept of Medical Microbiology, Immunology, and Cell Biology, Southern Illinois University School of Medicine, Springfield, Illinois, 62794, United States of America; 2 Dept of Pharmacology, Southern Illinois University School of Medicine, Springfield, Illinois, 62794, United States of America; 3 Simmons Cancer Institute, Southern Illinois University School of Medicine, Springfield, Illinois, 62794, United States of America; 4 Laboratory of Cell and Developmental Signaling, National Cancer Institute-Frederick, Frederick, Maryland, 21702, United States of America; University of Birmingham, UNITED KINGDOM

## Abstract

CLCA2 is a p53-, p63-inducible transmembrane protein that is frequently downregulated in breast cancer. It is induced during differentiation of human mammary epithelial cells, and its knockdown causes epithelial-to-mesenchymal transition (EMT). To determine how CLCA2 promotes epithelial differentiation, we searched for interactors using membrane dihybrid screening. We discovered a strong interaction with the cell junctional protein EVA1 (Epithelial V-like Antigen 1) and confirmed it by co-immunoprecipitation. Like CLCA2, EVA1 is a type I transmembrane protein that is regulated by p53 and p63. It is thought to mediate homophilic cell-cell adhesion in diverse epithelial tissues. We found that EVA1 is frequently downregulated in breast tumors and breast cancer cell lines, especially those of mesenchymal phenotype. Moreover, knockdown of EVA1 in immortalized human mammary epithelial cells (HMEC) caused EMT, implying that EVA1 is essential for epithelial differentiation. Both EVA1 and CLCA2 co-localized with E-cadherin at cell-cell junctions. The interacting domains were delimited by deletion analysis, revealing the site of interaction to be the transmembrane segment (TMS). The primary sequence of the CLCA2 TMS was found to be conserved in CLCA2 orthologs throughout mammals, suggesting that its interaction with EVA1 co-evolved with the mammary gland. A screen for other junctional interactors revealed that CLCA2 was involved in two different complexes, one with EVA1 and ZO-1, the other with beta catenin. Overexpression of CLCA2 caused downregulation of beta catenin and beta catenin-activated genes. Thus, CLCA2 links a junctional adhesion molecule to cytosolic signaling proteins that modulate proliferation and differentiation. These results may explain how attenuation of CLCA2 causes EMT and why CLCA2 and EVA1 are frequently downregulated in metastatic breast cancer cell lines.

## Introduction

Breast cancer relapse is due primarily to metastatic spread that occurs before or during treatment [1]. One of the body’s most potent defenses against metastasis is the anti-proliferative and anti-invasive signaling machinery based at cell-cell junctions. Adherens junctions (AJ) sequester beta catenin, a transcriptional activator of Myc and mesenchymal transcription factor genes that is upregulated in virtually all cancers [2, 3]. The loss of epithelial junctional markers during tumor progression is thought to occur by epithelial-to-mesenchymal transition, a process that at once releases cells from anchorage-dependence and confers invasiveness, resistance to chemotherapy, and stem-like properties [2, 4, 5]. EMT is suppressed by AJ protein E-cadherin, which sequesters beta catenin and inhibits mesenchymal transcription factors [6]. Attenuation of E-cadherin expression is sufficient to drive EMT in mammary epithelium, and E-cadherin is frequently mutated in invasive lobular cancers [2, 7].

The CLCA gene family arose in placozoans, the first multicellular organisms to develop epithelial tissues with organized cell-cell junctions [8]. In mammals CLCAs comprise four subfamilies [9]. They are distinguished by the juxtaposition of metalloprotease and VWA domains and the capacity to self-cleave [10]. CLCA2 is a type I integral transmembrane protein [11]. We recently demonstrated that CLCA2 is a stress-inducible gene, being strongly induced by p53 in response to cell detachment, DNA damage, and other stressors [12]. CLCA2 is frequently downregulated in breast cancers by promoter methylation, and ectopic expression in a breast cancer cell line inhibited tumor formation by tail vein injection and xenograft [13, 14]. In vitro, viral transduction inhibited proliferation of HMEC and induced apoptosis or senescence in breast cancer cells, while knockdown reduced mortality in response to the DNA damaging agent doxorubicin [12]. Consistent with an antiproliferative role for CLCA2, a recent study found that it was the most upregulated gene when AP1 oncogenic transcription factor was downregulated and that AP1 components Jun-1 and Fra-1 bound directly to the CLCA2 gene [15]. CLCA2 has also been reported to suppress migration and invasion in breast and colorectal cancer cell lines [14, 16].

CLCA2 is strongly associated with epithelial differentiation in breast and is downregulated in many breast cancers, most dramatically in the mesenchymal subtype [17]. CLCA2 is upregulated 150-fold when MCF10A HMEC reach confluency, which triggers mesenchymal-to-epithelial transition (MET) in that cell line [17, 18]. This association with MET was confirmed in another immortalized HMEC cell line, HMLE, which spontaneously undergoes MET to form cobblestone islands that express E-cadherin and other epithelial markers [4]. CLCA2 was upregulated in the islands [17,19]. Moreover, CLCA2 was downregulated in response to EMT induced by ectopic expression of mesenchymal transcription factors, TGF beta, or cell dilution [17].

Furthermore, we found that knockdown of CLCA2 by shRNAs provoked EMT in both MCF10A and HMLE, establishing that CLCA2 is a driver of epithelial differentiation rather than a passenger. Indeed, CLCA2 knockdown in HMEC caused focus formation, enhanced invasiveness, and increased mammosphere formation; these changes were accompanied by downregulation of E-cadherin and upregulation of mesenchymal markers [17].

To discover how CLCA2 promotes epithelial differentiation, we turned to a surrogate genetic system to search for interacting proteins. We screened cDNA libraries using a two-hybrid system designed for membrane-bound proteins (DualSystems). Although CLCA proteins have been proposed to be accessory proteins for chloride channels [20], the screen did not detect interactions with channels. Instead, one of the strongest interactions proved to be with Epithelial V-like Antigen 1 (EVA1), a Type I transmembrane protein whose ectodomain contains an Ig-like V-domain similar to that of Junctional Adhesion Molecules (JAMs). EVA1 is conserved throughout vertebrates but not beyond (http://useast.ensembl.org/Multi/GeneTree/Image?gt=ENSGT00640000091161). Like CLCA2, it is regulated by p53, p63, and p73 [21,22,23]. Genes with this regulatory profile are typically involved in terminal differentiation and cell adhesion [23]. EVA1 is thought to mediate homophilic cell-cell adhesion in diverse epithelial tissues, but it has not been studied in breast [24].

We show herein that 1) EVA1 is frequently downregulated in breast tumors and breast cancer cell lines, especially those of mesenchymal phenotype; 2) like CLCA2, EVA1 is an epithelial marker that is co-regulated with E-cadherin; 3) knockdown of EVA1 results in rapid EMT in immortalized HMEC; and 4) CLCA2 and EVA1 form a complex with junctional regulatory molecule ZO-1; and 5) that CLCA2 cytoplasmic tail binds to beta catenin. These findings indicate that EVA1 is a cell-cell junction component and that one role of CLCA2 in differentiation is to link cell-cell adhesion to homeostatic signaling.

## Materials and Methods

### Ethics Statement

The research described herein meets all applicable standards for the ethics of experimentation and research integrity. Human cancer data were derived from public databanks or cell lines that are exempt from IRB requirements. The cell line HMLE was a kind gift from Robert Weinberg (MIT). Its construction from anonymous, commercially available human mammary epithelial cells (Clonetics) has been published [4].

### Cell lines and cell culture

Breast cell lines MCF-10A, MCF-7, and MDA-MB-231 were obtained from ATCC in 2011 (Manassa, VA, USA) and grown in the media specified. HMLE is a human mammary epithelial line that was immortalized by hTERT [4]. It was a kind gift from Robert Weinberg (MIT, Boston, MA, USA) in 2010 and was grown as described [4]. Knockdown of EVA1 in HMLE and MCF7 was done by lentiviral transduction of small hairpin RNAs (GIPZ, OpenBiosystems). The two most effective out of 5 tested were termed EVA1sh7 and EVA1sh8 (clones #V3LHS_376099 and V3LHS_410864, respectively). Gipz-NC was used as a negative control. MCF-7 and MCF-10A cells were transduced with Myc-tagged EVA1 for localization experiments.

### Plasmid constructions

Human EVA1 having Myc and Flag-tags at its C-terminus in pCMV6-Entry Vector was purchased from Origene. The flag-tag was removed and EVA1-myc was transferred into pLEX-MCS vector by overlap extension PCR using the forward primer, 5’ to 3’, GAAGACACCGACTCTACTAGAGGATCCGGTAC CGAGGAGATCTGCCGC and reverse primer, 5’ to 3’, TTCCACCACACTGGACTAGTCTA GATATCATTTGCTGCCAGATC. Cells transduced with lentivirus were selected with 1 microgram/ml puromycin for a minimum of 2 weeks before performing RNA and protein analysis. For co-expression of EVA1 and CLCA2, EVA1 was inserted into pLenti-GFP-Hygro (Addgene). HEK293 cells were first transduced with pLex-CLCA2, then pLenti-Hygro-EVA1, then selected with 1micrograms/ml puromycin and 50 micrograms /ml hygromycin. CLCA2 deletion mutants were constructed using PCR and other standard molecular methods. Replacement of the CLCA2 ectodomain with Myc-tagged GFP was described previously [11]. Additional variations adding the cytoplasmic tail or substituting the EVA1 TMS (residues 155–180) were constructed by PCR and all were transferred to pLex. A lentivirus expressing CLCA2 from a PGK promoter was constructed by inserting a cassette into the BamHI and KpnI sites of pLKO.1. The cassette encoded puromycin resistance, mCherry, and hCLCA2 linked by T2A and P2A segments.

### Bioinformatics

Oncomine (Compendia Bioscience, Ann Arbor, MI, USA), Gene Expression Omnibus (NCBI), NextBio, the Cancer Cell Line Encyclopedia (Broad-Novartis Institute), and curated data from The Cancer Genome Atlas were used to analyze the expression patterns of EVA1 in immortalized breast cell lines vs. breast cancer cell lines and breast tissues vs. breast tumor tissues.

### RNA and RT-qPCR analysis

Cells were grown to confluency except where noted and harvested using Trizol (Invitrogen). RNA was extracted and reverse transcribed as described [25, 12]. Expression was quantified by qPCR using an ABI7500 instrument. Primer sequences are available upon request.

### Western blot analysis

For protein analysis, cleared NP40 lysates and whole cell SDS lysates were prepared from 10-cm dishes containing cells at 100% confluency as described [17, 26]. Protein concentration was measured by BCA assay and 50 micrograms protein was loaded per lane. Immunoprecipitations were performed as described except that the buffer contained 0.5% NP40 [11]. Antibodies for E-cadherin, N-cadherin, beta catenin, phospho-beta catenin, beta four integrin, and fibronectin were all rabbit polyclonal from Cell Signaling; for Myc-tag (clone 9E10.3), from Neomarkers; for FLAG tag (clone M2), Agilent; for actin (125-ACT), from PhosphoSolutions; for GFP, from Abcam; for vimentin, from Millipore. ZO-1 antibody was from PTG. The CLCA2 antibody TVE20 has been described (12). The protein size marker was Dual color (Bio-Rad). Secondary antibodies were labeled with IR680 or IR800 (Licor), and protein expression was quantified on an Odyssey instrument (Licor).

### Immunofluorescence and confocal analysis

MCF7 cells stably transduced with EVA1-myc were grown on poly-L-lysine coated coverslips for 8 to 10 days. The cells were fixed with cold methanol, and blocked with blocking buffer (1% FBS and 1% BSA in PBS) overnight at 4C. The cells were then incubated with the same primary antibodies for E-cadherin and ZO-1 used for immunoblots. EVA1 was detected using anti-Myc 9E10. This was followed by secondary antibodies labeled with AlexaFluor 568 or AlexaFluor 488 (Invitrogen) according to manufacturers’ recommendations. Nuclei were stained with 4,6-diamidino-2-phenylindole (DAPI). Coverslips were mounted onto slides using ProLong Gold mounting medium (Invitrogen). The cells were visualized under a fluorescent compound microscope prior to confocal laser-scanning microscopic analysis (Leica LAS-TF). The cells were scanned for Z-stacks from basal to apical side. Images were collected from XYZ-axes with each section thickness 1μm. Orthogonal images were obtained using the Leica software in XY and XZ planes to analyze the spatial localization of junctional proteins. Transduced HEK-293 cells were grown to superconfluency and treated similarly. We found that high cell density was necessary for junctional maturation and apical localization of ZO-1 in this cell line. MCF10A cells were stained with anti-CLCA2 antibody (Sigma Prestige, #HPA47192) and examined using an inverted microscope (Olympus IX71).

### Statistics

Each experiment was repeated three times and Student’s t-test was performed to determine the statistical significance of the results. P values less than 0.05 were considered significant.

## Results

### Interaction of CLCA2 and EVA1

**Fig 1:**
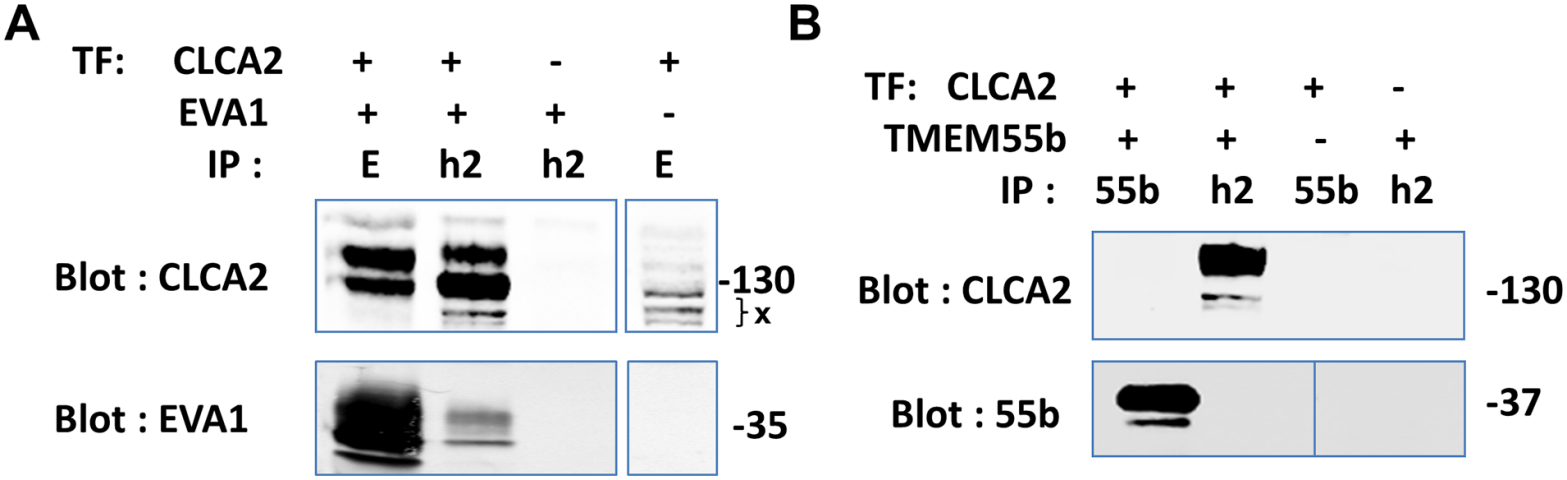
Co-immunoprecipitation assay of two-hybrid interactors. Flag-tagged candidate cDNAs were transfected into HEK293T cells with or without CLCA2 cDNA and lysates were subjected to co-immunoprecipitation and immunoblot with antibodies specific for FLAG or CLCA2. The CLCA2 antibody was TVE20. A, EVA1 was precipitated by CLCA2 antibody only from cells transfected (TF) with both. X, nonspecific bands. B, TMEM55b failed to co-precipitate. E, EVA1; h2, human CLCA2; 55b, TMEM55b. Vertical lines indicate omitted lanes. Sizes marked in kilodaltons.

To determine how CLCA2 promotes MET, we conducted a two-hybrid screen using a split-ubiquitin system designed for transmembrane proteins, wherein bait and prey are expressed in the yeast endoplasmic reticulum (DualSystems Biotech, Basel, Switzerland). Putative interactors from the yeast screen were further tested by co-transfection into 293T cells followed by co-immunoprecipitation (co-IP) assays. Candidate proteins were labeled with C-terminal FLAG tags. We found that EVA1, which ran as a cluster of three diffuse bands, could be precipitated by CLCA2 antibody from co-transfected cells but not from cells transfected with EVA1 alone (Fig 1A). Similarly, CLCA2 was precipitated with FLAG antibody from co-transfected cells but not from cells transfected with CLCA2 alone. A second two-hybrid interactor, TMEM55b, did not interact by co-IP and thus served as an additional negative control (Fig 1B).

### CCA2 binds EVA1 via its transmembrane segment

**Fig 2:**
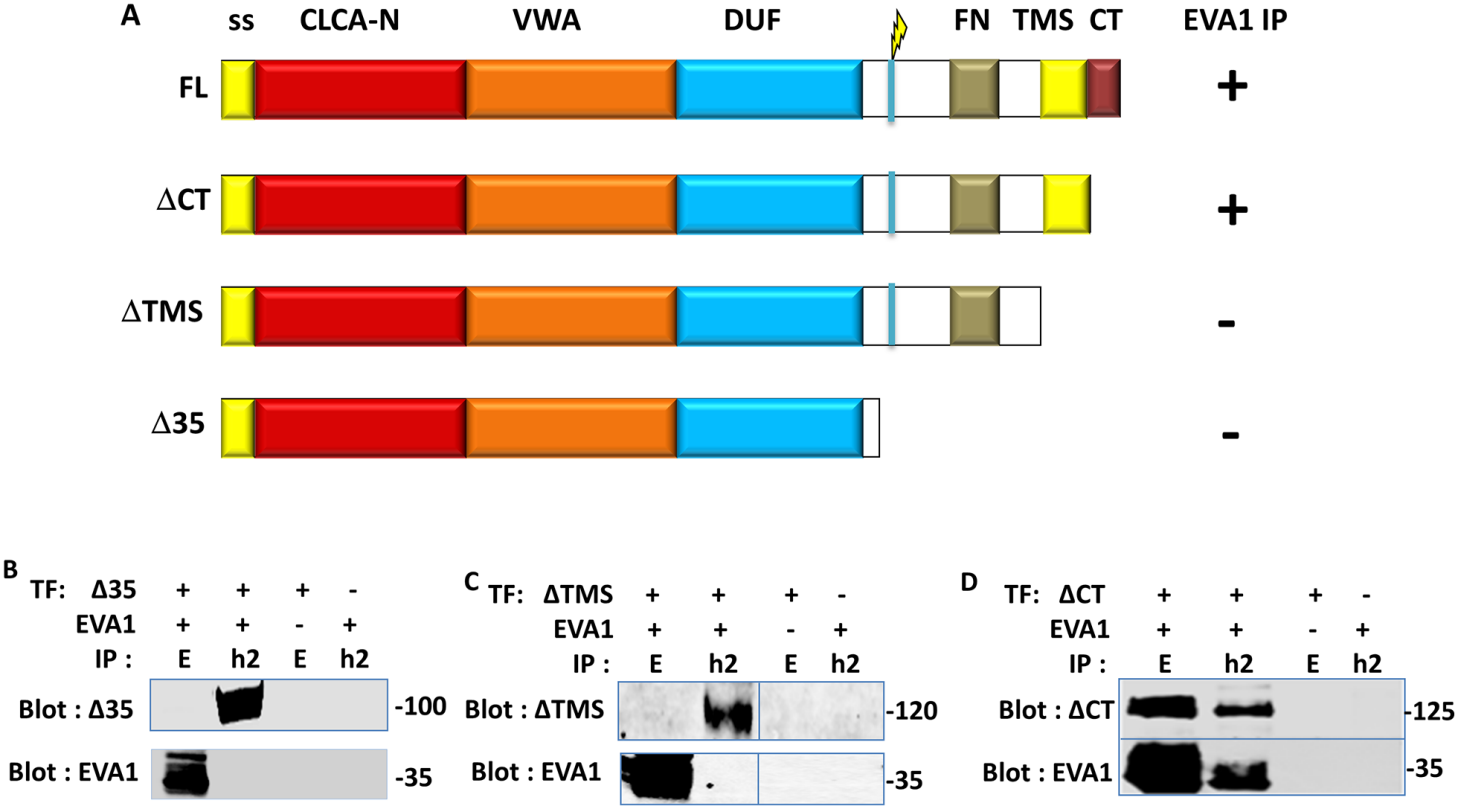
Identification of the CLCA2 domain that interacts with EVA1. A. constructs used to identify the CLCA2 domain that interacts with EVA1. CLCA2 interaction with EVA1 is denoted by + or - after each construct. B-D, immunoprecipitation from co-transfected cells indicates requirement for the transmembrane segment (TMS). EVA1 was detected with Flag antibody and CLCA2, with TVE20.

**Fig 3:**
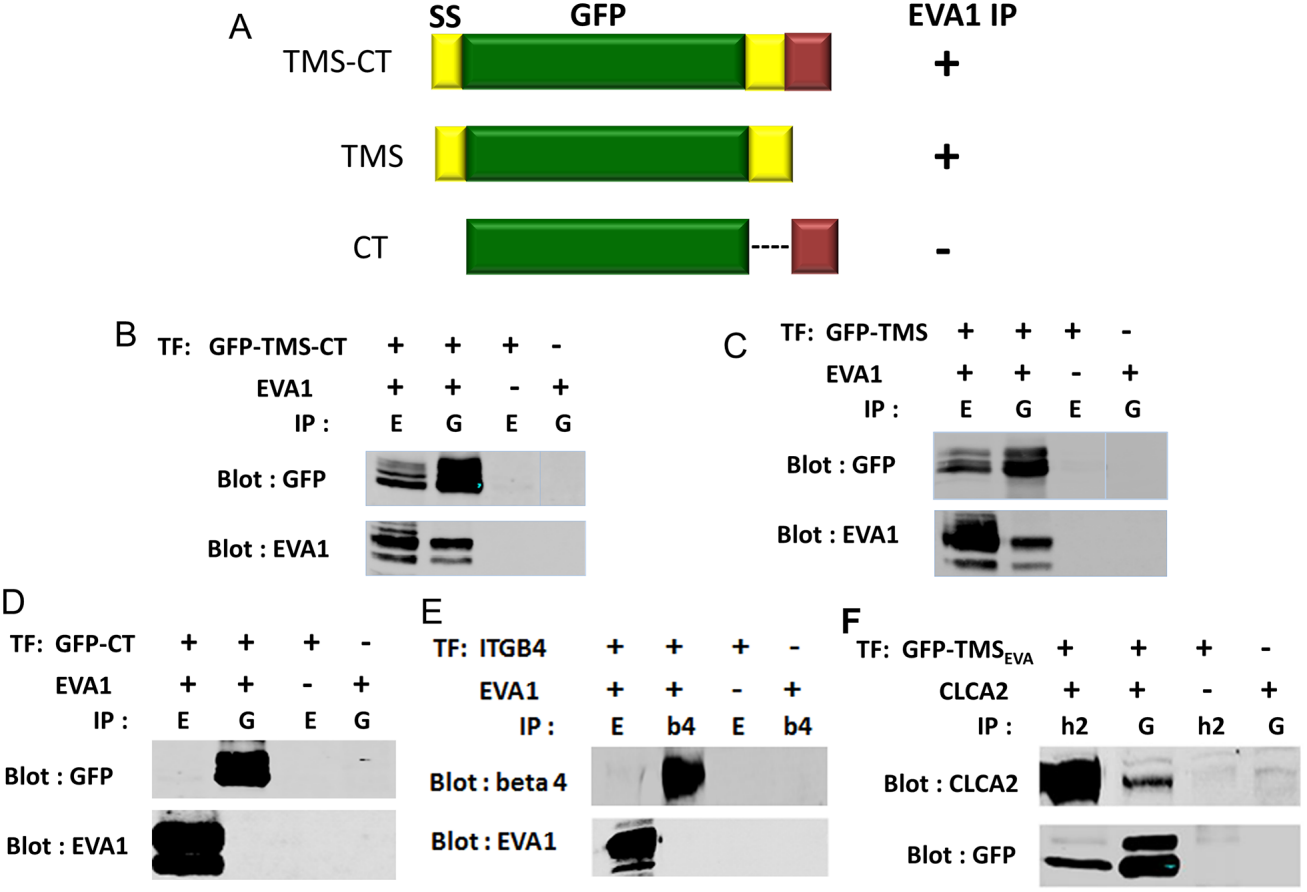
Confirmation of the TMS as the interacting domain. A, substitution of GFP for the CLCA2 ectodomain with CLCA2 C-terminal domains appended. CLCA2 interaction with EVA1 is denoted by + or - after each construct. B- D, immunoprecipitation from cells co-transfected with constructs and EVA1 indicates that CLCA2 TMS is required but not cytoplasmic tail. E, immunoprecipitation from cells co-transfected with EVA1 and integrin beta four. F, immunoprecipitation from cells co-transfected with ssGFP tagged with the EVA1 TMS. ssGFP by itself failed to interact with either CLCA2 or EVA1 (not shown). E, EVA1. G, GFP. Antibodies: EVA1, Flag; GFP constructs, GFP; CLCA2, TVE20.

To gain insight into the purpose of the interaction, we asked which domain of CLCA2 interacted with EVA1. Testing of deletions encompassing the region C-terminal to the cleavage site, the transmembrane segment (TMS), or the cytoplasmic tail (CT) suggested that the TMS was responsible (Fig 2B and 2C). Alternatively, removing the TMS might have merely disrupted the plasma membrane localization of the protein, preventing the interaction. To test this possibility, the ectodomain was replaced with GFP (Fig 3A and 3B). A strong co-IP was still observed. Removal of the CT had no effect on the interaction, and EVA1 failed to interact when the CT alone was fused to GFP, ruling out a role for that domain (Fig 3C and 3D). These results confirm that EVA1 interacts with the TMS of CLCA2. To rule out the possibility that EVA1 might interact indiscriminately with any TMS, EVA1 and integrin beta four were co-transfected and immunoprecipitated. No interaction was seen (Fig 3E).

**Fig 4:**
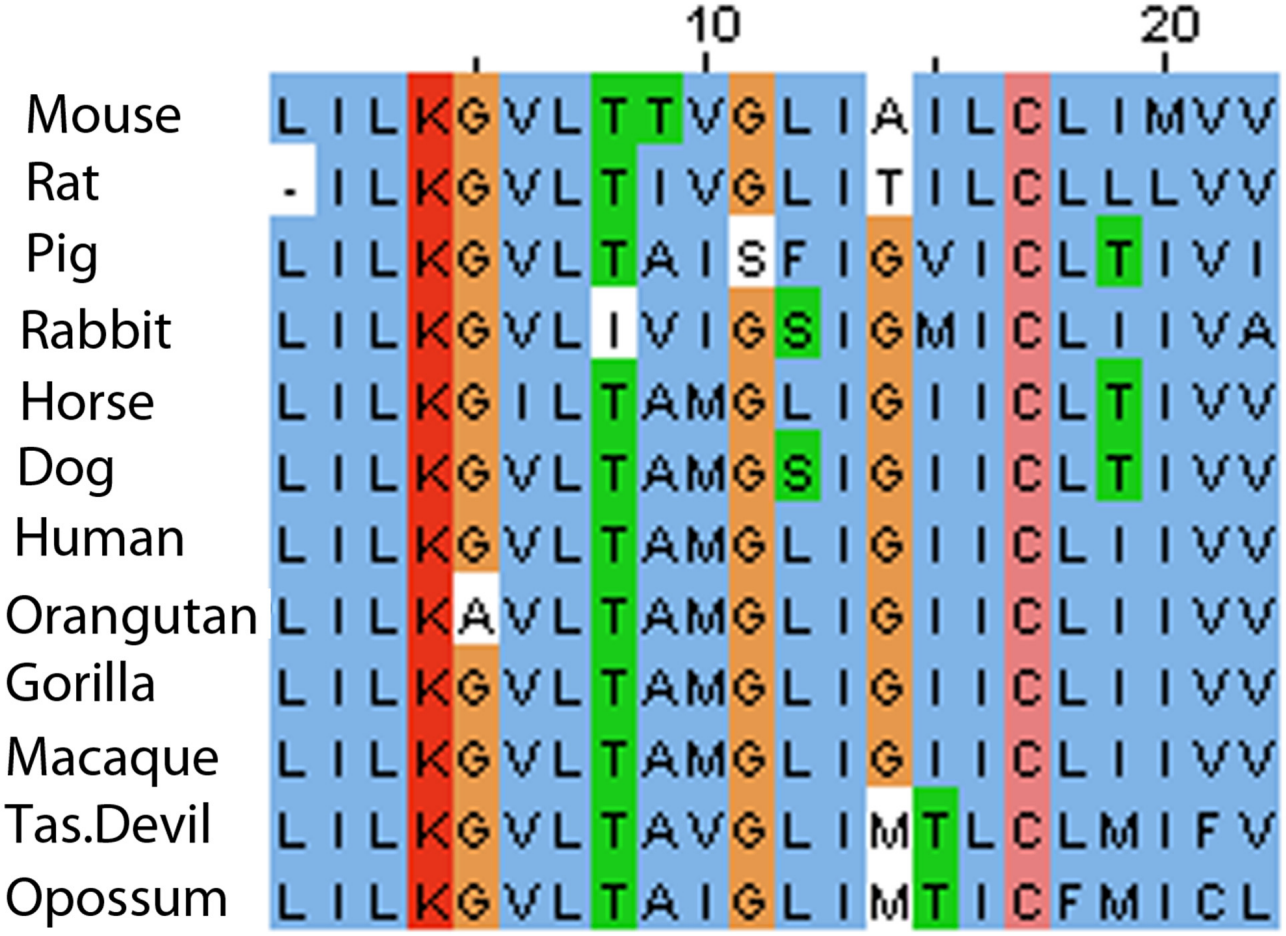
Conservation of CLCA2 TMS amino acid sequence in mammals. Six positions were conserved in all mammals tested and five diverged in only one species. Dissimilar substitutions are boxed. Data from UNIPROT were aligned by ClustalW, JALview.

As the interaction with EVA1 must occur within the membrane bilayer, the TMS of EVA1 was considered most likely to interact with that of CLCA2. Accordingly, appending the EVA1 TMS to ssGFP allowed it to co-IP with CLCA2 (Fig 3F). Alignment of the TMS segments of mammalian CLCA2 homologs revealed that the sequence of the TMS is highly conserved, especially the lysine, cysteine, and three glycine residues, suggesting a function beyond anchoring the protein in the lipid bilayer (Fig 4). The other human homologs, CLCA1 and CLCA4, lack this sequence, suggesting that interaction with EVA1 is intrinsic to CLCA2.

### EVA1 is an epithelial marker that is downregulated in breast cancer EMT

**Fig 5:**
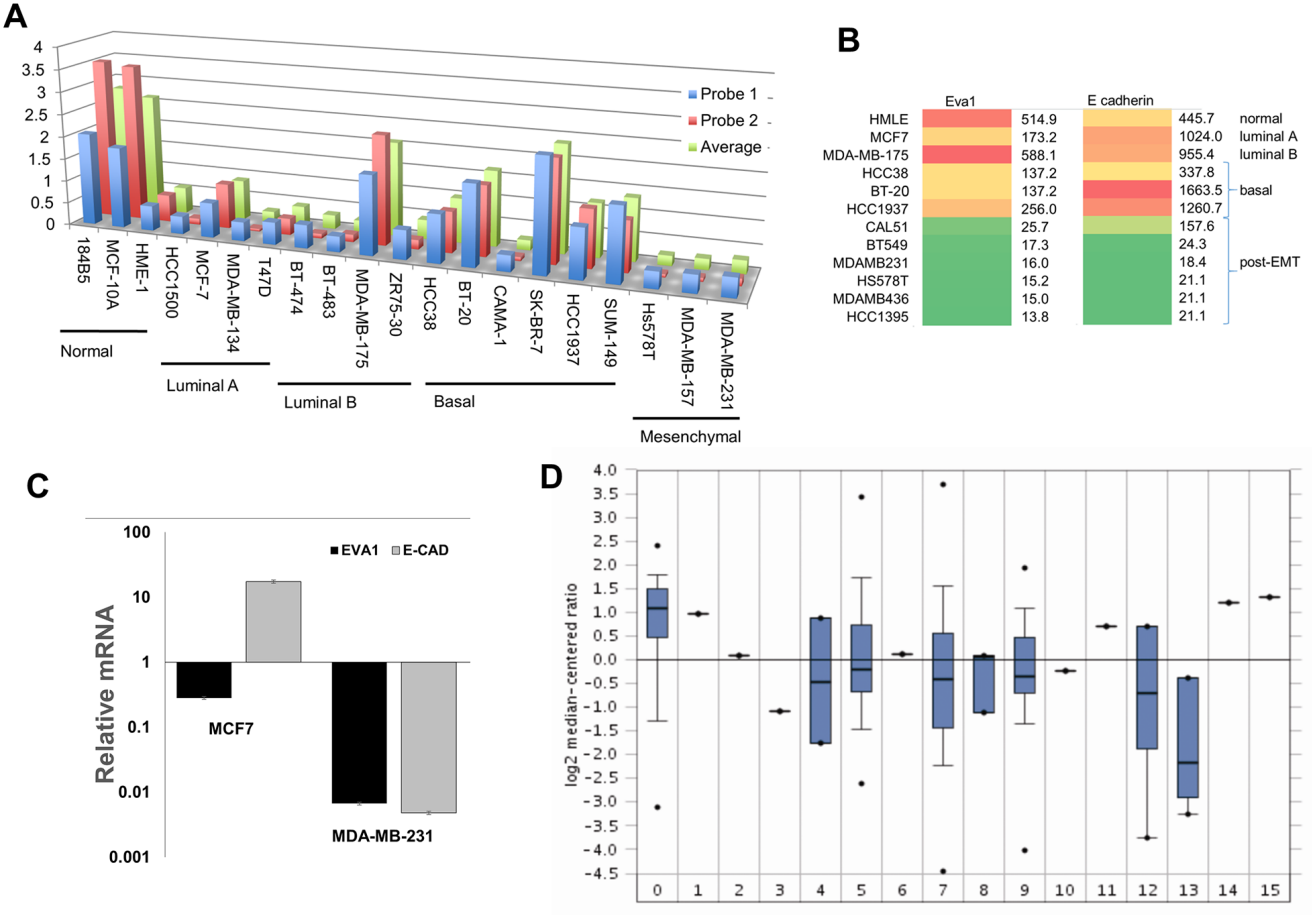
EVA1 downregulation in breast cancer. A, EVA1 gene expression profiles extracted from a study of breast cell lines [59] using GEO. B, heat-maps comparing EVA1 with E-cadherin expression in breast cell lines using data from the Cancer Cell Line Encyclopedia (3). Green indicates lowest expression and red indicates highest. C, expression profiles by qPCR showing downregulation of EVA1 and E-cadherin in the mesenchymal cell line MDA-MB-231 relative to luminal MCF7. Values were normalized to levels in immortalized HMEC, HMLE. p <0.05 for all pairwise comparisons. D, breast cancer expression profiles extracted from The Cancer Genome Atlas (TCGA1) databank using Oncomine. The log2 median-centered ratios are depicted in box-and-whisker plots. Dots represent maximum and minimum outliers from the main dataset. For each plot, the following pathological subtypes were evaluated separately. 0, normal tissue (61); 1, apocrine carcinoma (1); 2, large cell neuroendocrine (1); 3, ductal carcinoma (1); 4, intraductal cribriform adenocarcinoma (3); 5, invasive carcinoma (76); 6, invasion cribriform carcinoma (1); 7, invasive ductal carcinoma (395); 8, invasive ductal and lobular carcinoma (3); 9, invasive lobular breast carcinoma (36); 10, invasive papillary breast carcinoma (1); 11, metaplastic breast carcinoma (1); 12, mixed lobular and ductal breast carcinoma (7); 13, mucinous breast carcinoma (4); 14, papillary carcinoma (1); 15, pleomorphic carcinoma (1). Number in parentheses indicates sample size for each category.

By searching public databases, we found that EVA1 was frequently downregulated in breast cancer cell lines with mesenchymal expression profiles compared to cell lines that retained epithelial markers, such as some luminal and basal (Fig 5A). We obtained similar results using the Cancer Cell Line Encyclopedia dataset [3] and further observed that EVA1 expression level correlated with that of junctional marker E-cadherin (Fig 5B). This was confirmed by RT-qPCR with representative cell lines (Fig 5C).

To determine EVA1 status in breast tumors, we queried the TCGA breast cancer dataset using Oncomine (Fig 5D). We found that EVA1 expression was often downregulated in breast tumors (lanes 1–15) relative to normal breast tissue (lane 0). As above, tumor types with high median expression of EVA1 also had high expression of E-cadherin (data not shown). It should also be noted that EVA1 is expressed in some lymphocytes [27] and arterial endothelial cells [28], so that immune infiltration or tumor vascularization could inflate apparent expression in tumor samples.

These results paralleled the behavior of CLCA2 and suggested that EVA1 might also be a marker of epithelial phenotype. To test this, we used Oncomine and NextBio to consult transcriptional profiles from cell culture systems that model mammary epithelial differentiation. In the first study, primary human mammary epithelial cells were cultured in three-dimensional Matrigel to promote epithelial differentiation or grown as mammospheres to promote stem-like properties [29]. Expression of EVA1 and CLCA2 was seven to eightfold higher in the Matrigel population (Table 1). In the second study, the immortalized mammary epithelial cell line MCF10A was cultured in monolayer on plastic or on permeable membranes that support normal apico-basal polarization, barrier formation, and other aspects of differentiation [30]. EVA1 expression was threefold higher and CLCA2 was seven times higher in the differentiated population. On the other hand, the genes were downregulated in parallel when EMT was induced by TGF beta, expression of mesenchymal transcription factors, knockdown of E-cadherin [31], or ectopic expression of miR221/222 [32].

**Table 1:** Association with epithelial differentiation.

Differentiation Cue	Fold Change in mRNA	
	EVA1	CLCA2
**Matrigel vs. mammospheres**^1^	7.23 +/- 2.77	7.74 +/- 3.98
**Barrier establishment**^2^	2.95	6.98
**TGF beta**^3^	-11.4	-2.7
**SNAIL 1**^3^	-8.94	-3.78
**Goosecoid**^3^	-15.7	-8.73
**E-Cadherin KD**^3^	-3.48	-3.47
**TWIST 1**^3^	-7.2	-5.08
**E-Cadherin/beta-catenin KD**^3^	-50.7	ND
**Mir-221/mir-222**^4^	-4.6	-4.6

^1^Average of 2 experiments +/- standard deviation

^2-4^P < 0.05

**Fig 6:**
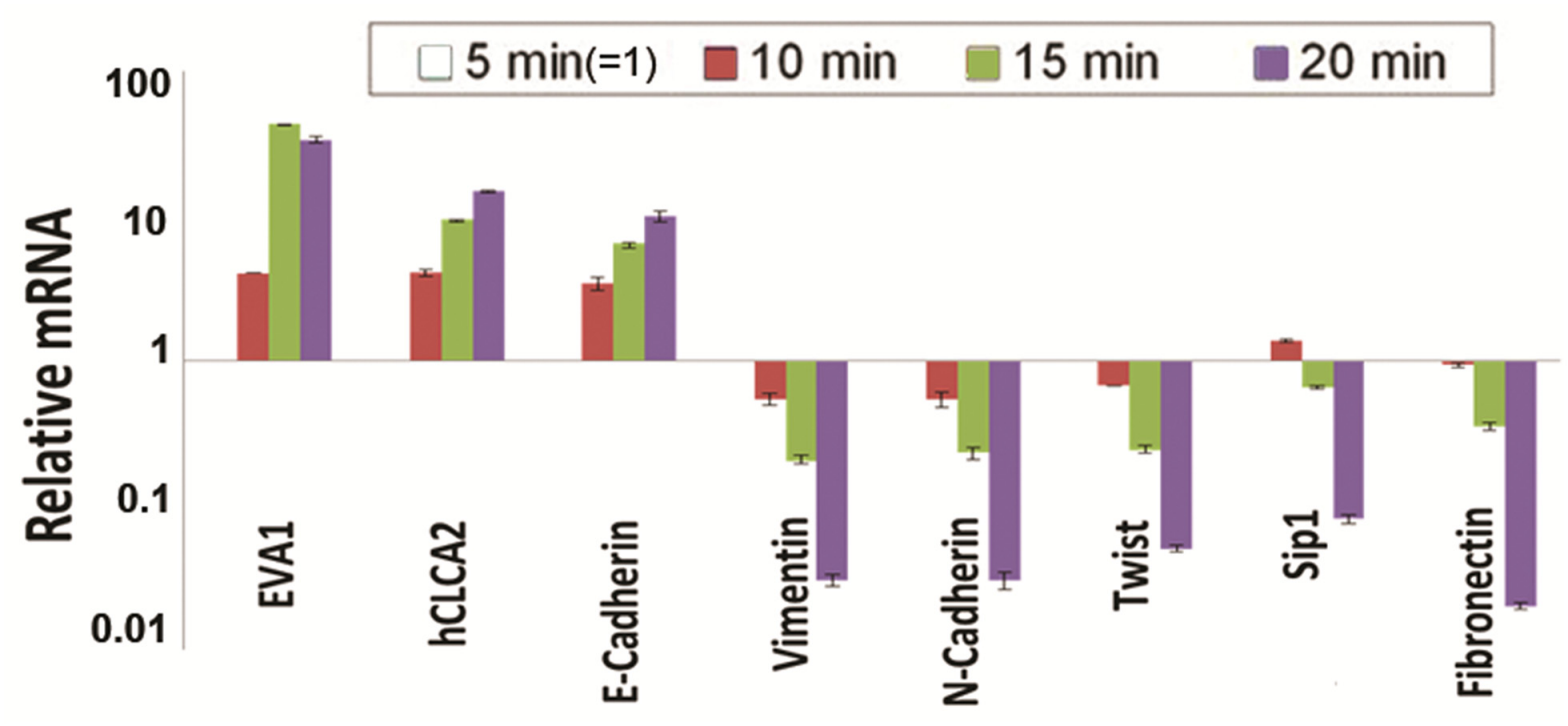
Expression of EVA1 correlates with epithelial differentiation. HMLE cells were separated into epithelioid and mesenchymal subpopulations by differential trypsinization and subjected to RT-qPCR. The transcriptional profile reveals that EVA1 is highest in the most trypsin-resistant fraction, correlating with E-cadherin and CLCA2. The 5min fraction was normalized as 1. P<0.01 for all comparisons between 5min and 20min samples.

We confirmed the association of EVA1 with epithelial phenotype using the mammary epithelial cell line HMLE, which undergoes spontaneous MET with increasing cell density, forming cobblestone-like islands with high expression of E-cadherin and other junctional markers [4]. The islands can be separated from surrounding mesenchymal-like cells by differential trypsinization [19]. Transcriptional profiling of the fractions by RT-qPCR revealed that EVA1 correlates directly with E-cadherin but inversely with mesenchymal markers (Fig 6). These results indicate that EVA1 is an epithelial marker whose expression is lost upon EMT.

### EVA1 knockdown triggers EMT

**Fig 7:**
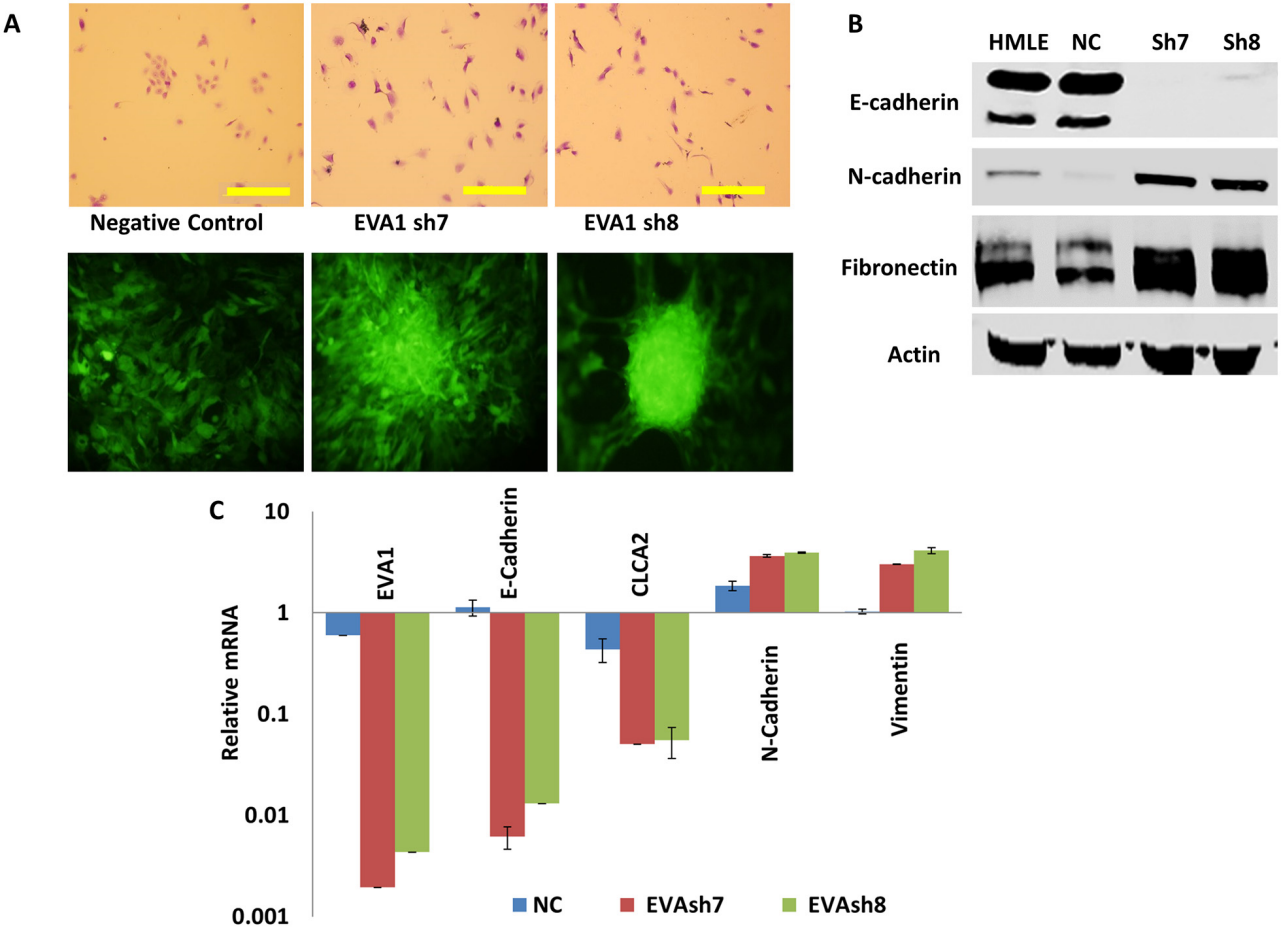
Knockdown of EVA1 induces EMT. HMLE cells were lentivirally transduced with EVA1 shRNAs sh7 and sh8 or non-silencing control GipzNC. **A**, phase-contrast (top) and fluorescence micrographs showing changes in cell behavior after knockdown. Top was stained with crystal violet, bottom shows GFP. Scale bar, 200 microns. **B**, immunoblots of whole cell lysates probed for EMT marker proteins. **C**, transcriptional profile showing attenuation of EVA1 and EMT in knockdown cell lines. Values were normalized to HMLE parent. P<0.01 for each pairwise comparison between GipzNC and EVAsh7 or EVAsh8.

Cell junctional adhesion proteins such as E-cadherin mediate signaling pathways that maintain epithelial differentiation, and their downregulation leads to EMT and tumorigenesis [2,4]. We supposed that EVA1, as a junctional protein, might also promote epithelial differentiation and that CLCA2 might promote epithelial integrity by regulating EVA1 localization or activity. To test this hypothesis, we knocked down EVA1 in HMLE using two lentiviral constructs, sh7 and sh8 (Gipz, OpenBiosystems). Both caused a change to mesenchymal morphology, loss of cell clustering at low density, and focus formation at high density (Fig 7A). Transcriptional and protein profiling confirmed EVA1 knockdown and a shift from epithelial to mesenchymal phenotype (Fig 7B and 7C). These results revealed that EVA1 is required for epithelial phenotype in vitro.

### Co-Immunolocalization of EVA1 and CLCA2

**Fig 8:**
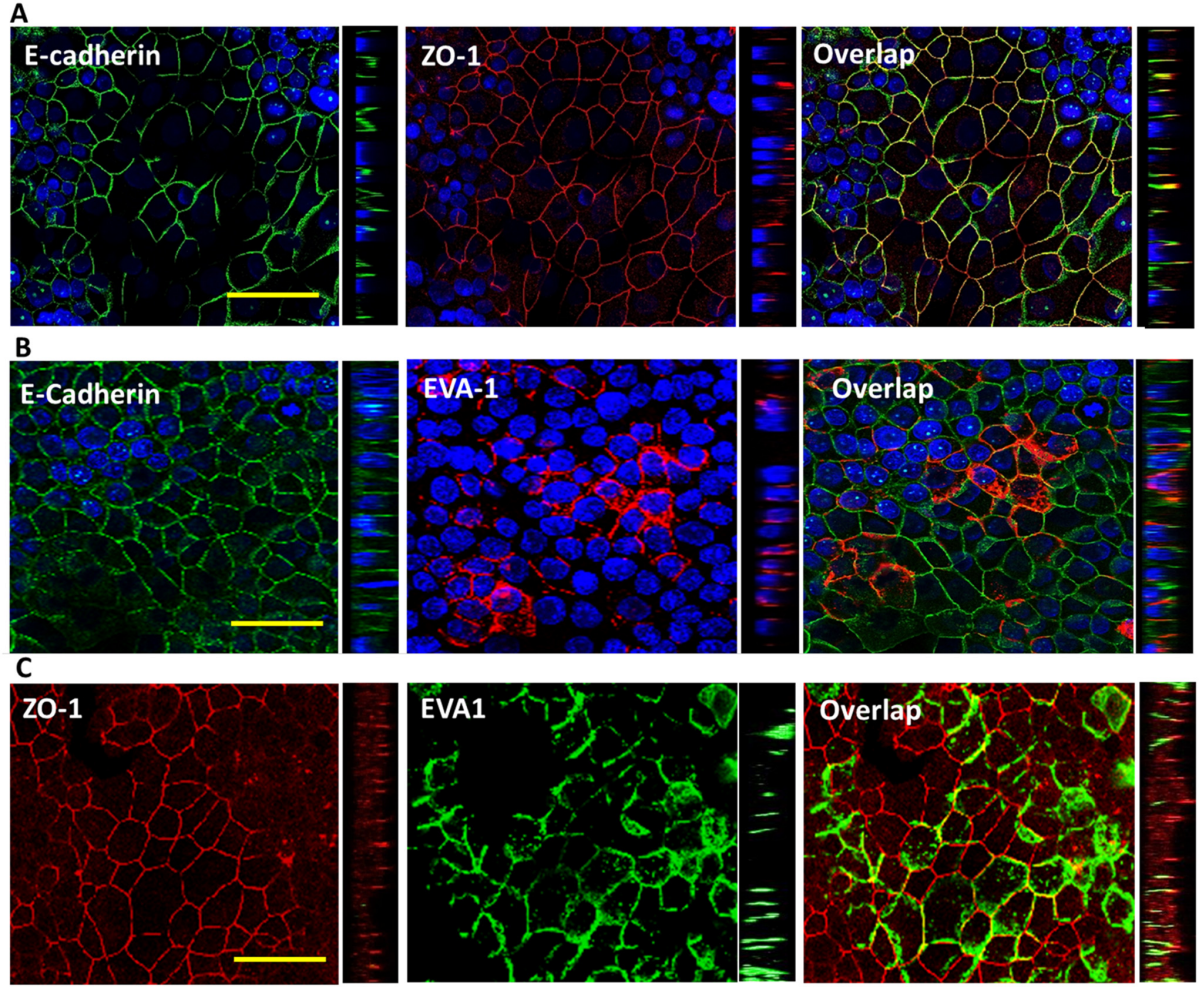
EVA1 localizes throughout lateral interfaces of cell-cell junctions. Confocal micrographs of MCF7-EVA1 cells grown to high density and probed for A, E-cadherin and ZO-1; B, E-cadherin and EVA1; or C, ZO-1 and EVA-1. In A and B, nuclei were stained with DAPI (blue). To the right of each XY image is a YZ image showing that EVA1localization resembles that of E-cadherin rather than ZO-1. Scale bar, 50 microns.

The structure of EVA1 resembles that of Junctional Adhesion Molecules (JAMs) which reside at tight junctions. To determine its localization, we transduced EVA1 into a breast cancer cell line known to form tight junctions, MCF7, and performed immunofluorescence and confocal microscopy. As expected, the cells showed apicolateral localization of the tight junction marker ZO-1 and lateral localization of E-cadherin (Fig 8A). Surprisingly however, EVA1 was found throughout the lateral junction like E-cadherin rather than being apical (Fig 8B and 8C).

**Fig 9:**
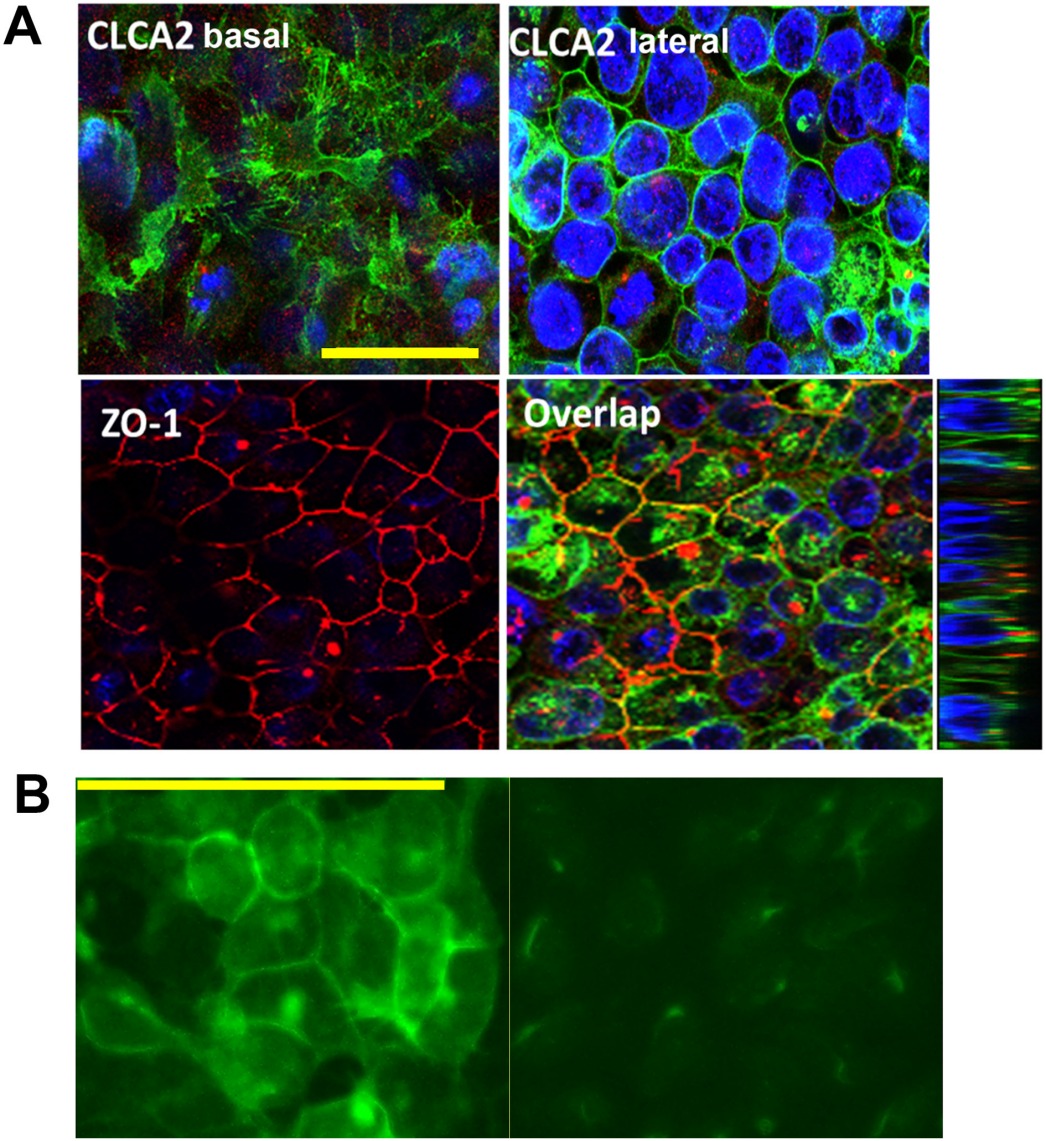
CLCA2 localizes to basal and lateral interfaces of cell junctions. **A**, confocal images of HEK293-CLCA2 cells grown to superconfluency and probed for CLCA2 or ZO-1. Top left, a basal plane from the Z-stack; top right, a central plane. Lower right, YZ image shows ZO-1 at apex of lateral junction, while CLCA2 occurs throughout junction. **B**, detection of endogenous CLCA2 in MCF10A (left) but not in cells expressing CLCA2 shRNA (right). Scale bars, 50 microns.

To determine CLCA2 localization, we initially used HEK293 cells because MCF7 cells do not tolerate its constitutive expression [12]. Unlike CLCA1 and CLCA4, its C-terminus contains a conserved match for the AP1-mediated basolateral sorting signal, D/ExxxLL [33]. Immunolocalization of HEK293 cells transduced with FLAG-tagged CLCA2 confirmed that CLCA2 is found both in the basal membrane and lateral junctions (Fig 9A). The junctional labeling was observed only in cells that had been confluent for several days and started to form multiple cell layers. In non-confluent cells, CLCA2 was found mostly in cytoplasmic vesicles.

To determine localization of endogenous CLCA2 in MEC, we used a novel antibody to perform IF on confluent MCF10A and knockdown controls (Fig 9B). A strong signal was seen at cell-cell junctions in wildtype but not knockdown cells.

**Fig 10:**
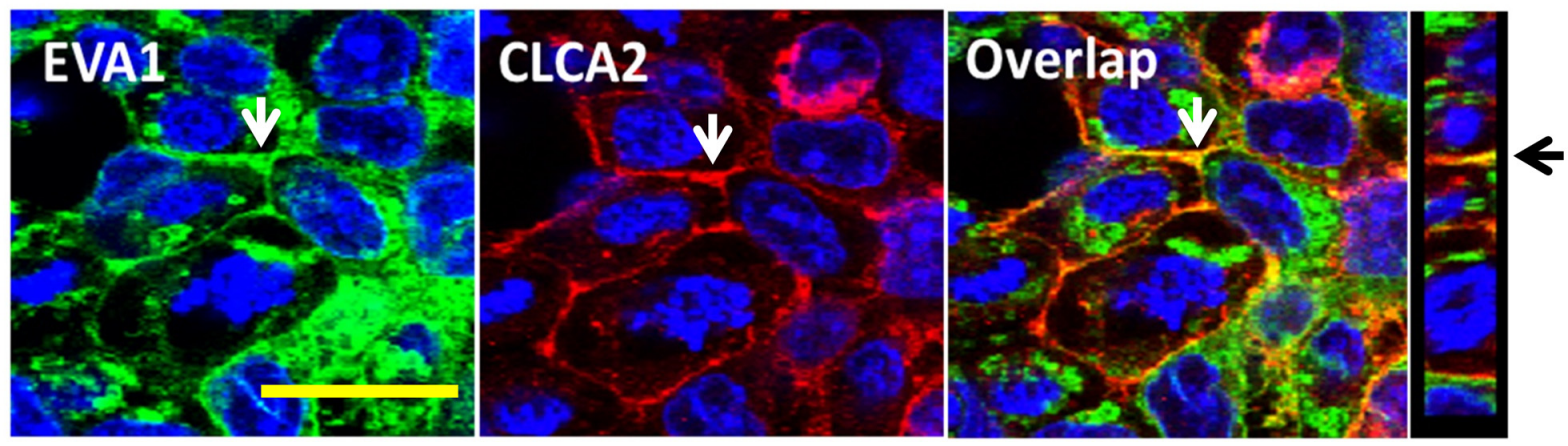
CLCA2 and EVA1 co-localize at cell-cell junctions. Confocal images of HEK293 cells stably expressing CLCA2-flag and EVA1-myc and stained for EVA1 (green), CLCA2 (red), and nuclei (blue). To the right is a YZ image. Arrows denote junctional staining. Scale bar, 50 microns.

To determine whether CLCA2 and EVA1 co-localize, HEK293 cells were co-transduced with both EVA1 and CLCA2 and grown to high cell density. We observed overlapping junctional localization, along with cytoplasmic expression (Fig 10, arrows).

### CLCA2 and EVA1 interact with junctional signaling molecules

**Fig 11:**
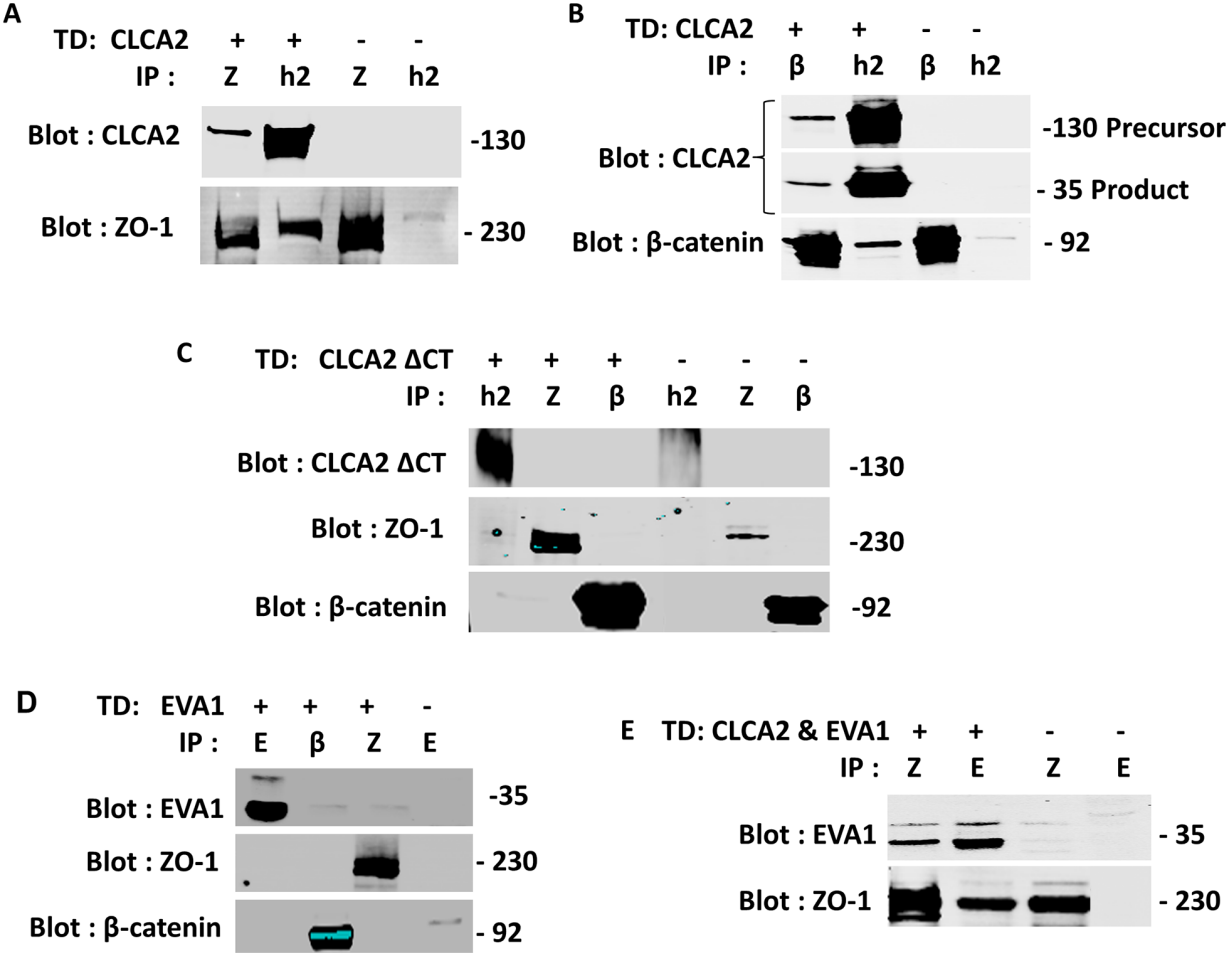
CLCA2 interaction with ZO-1 and β-catenin. **A** and **B**, HEK293 cells with or without stable CLCA2 expression were grown to superconfluency, and lysates were subjected to immunoprecipitation and immunoblot with antibodies specific for either CLCA2 (h2), ZO-1(Z) or β-catenin (β). **C**, cells expressing CLCA2 lacking the cytoplasmic tail were analyzed identically; no interactions were observed. **D**, failure of transduced EVA1 to co-IP with ZO-1 or beta catenin. **E**, co-IP of EVA1 with ZO-1 when CLCA2 was co-expressed. Except in **C**, CLCA2 contained a FLAG tag and EVA1 contained a Myc tag. In **C**, TVE20 antibody was used.

To determine how these proteins affect junctional signaling, we tested whether EVA1 or CLCA2 interacted with known components of TJ and AJ by co-IP from cells expressing either protein. Most potential interactors were negative, including E-cadherin, occludin, Zonab, and claudins (data not shown). However, CLCA2 interacted with signaling molecules from both TJ and AJ, ZO-1 and beta catenin (Fig 11A and 11B). Deletion of the cytoplasmic tail of CLCA2 abolished the interaction (Fig 11C). ZO-1 is an adapter protein that links TJ and AJ adhesion molecules to the cytoskeleton and is required for maturation of AJ [34]. This suggests that the CLCA2-EVA1 interaction links cell-cell adhesion by EVA1 to both TJ and AJ signaling pathways.

To determine whether CLCA2 allowed EVA1 to interact with ZO-1 and beta catenin, cells were transduced with both CLCA2 and EVA1, and co-IP studies were performed. We found that EVA1 co-purified with ZO-1 only in the presence of CLCA2 (Fig 11D and 11E). However, beta catenin did not co-IP with EVA1 (data not shown), suggesting that CLCA2 forms two independent complexes, one with EVA1 and ZO-1, the other with beta catenin and perhaps other adherens junction proteins.

### CLCA2 downregulates beta-catenin target genes

**Fig 12:**
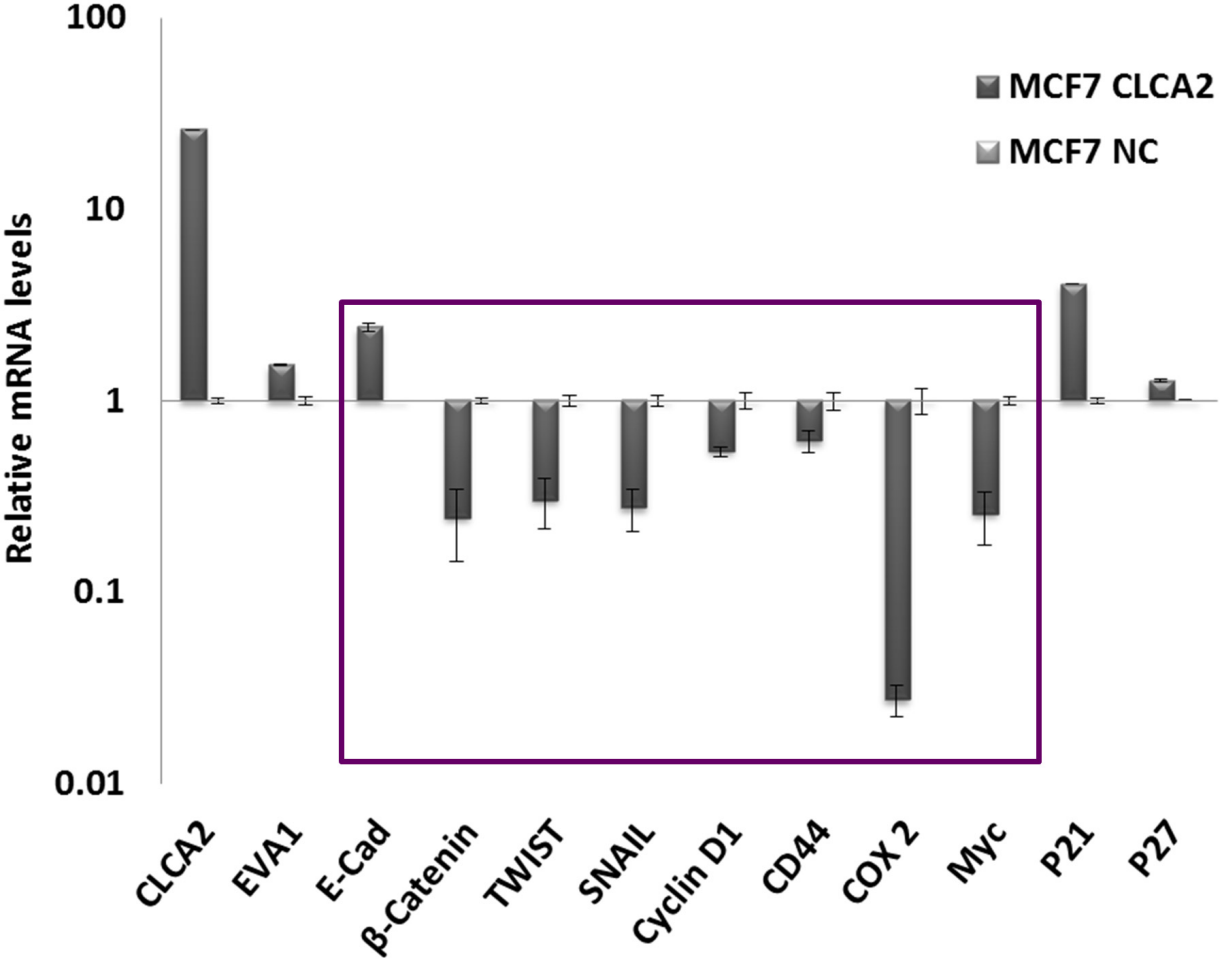
CLCA2 downregulates beta catenin-regulated genes. Transcriptional profile of beta catenin-client genes (boxed) in MCF7 cells engineered to moderately express CLCA2. Values are normalized to vector-transduced control (NC). P<0.05 for comparisons between MCF7-CLCA2 and control within the boxed set.

To determine whether CLCA2 affected beta catenin positively or negatively, we constructed a lentivirus expressing CLCA2 from a weak promoter to reduce toxicity and transduced it into MCF7 cells. Expression profiling of selected cells revealed downregulation of beta catenin target genes such as Cox2, Myc, and mesenchymal transcription factors such as Twist1 and Snail1 (Fig 12). These results suggest that CLCA2 is a negative regulator of beta catenin, the principal driver of EMT and effector of Wnt signaling. Furthermore, they may explain why ectopic expression of CLCA2 inhibits proliferation or migration in many cell types and knockdown triggers EMT in HMEC [14, 16, 17]. In contrast, transduction of EVA1 alone into MCF10A or MDA-MB231 had no apparent effect on proliferation or migration (data not shown). The former has high expression of both genes while the latter lacks both. The lack of effect of EVA1 overexpression in either cell line is consistent with a scenario in which EVA1 promotes differentiation through CLCA2, perhaps by regulating its localization or activity. Endogenous EVA1 already fulfills that role in MCF10A, so further EVA1 overexpression has no effect. On the other hand, the lack of CLCA2 in MDA-MB231 deprives EVA1 of the tool by which it promotes MET, so no differentiative effect is observed.

## Discussion

Stable cell-cell junctions are required for maintaining epithelial homeostasis, a primary defense against invasion and metastasis. Attenuation of E-cadherin or ZO-1 causes not only loss of junctional anchoring but triggers global programmatic changes that abet invasion and metastasis [4, 34]. Having observed similar changes in response to knockdown of CLCA2, we sought to understand the mechanism by using surrogate genetics. These studies revealed interaction with cell-cell adhesion protein EVA1 and unexpected interactions with the same signaling interface as E-cadherin, while no direct interaction with E-cadherin was found.

EVA1 was originally characterized as an epithelial cell-cell adhesion protein with a structure similar to that of tight-junctional JAMs [24]. However, it is best known from studies of the choroid plexus, which forms the barrier between the blood and cerebrospinal fluid [27, 35]. There, EVA1 is expressed by epithelial cells whose tight junctions restrict the passage of T cells into the CNS. Rag-/- mice lacking T-cells were shown to have increased permeability at the choroid plexus, but treating these mice with an activating antibody that recognizes EVA1 ectodomain increased expression and junctional localization of E-cadherin and reduced permeability of the choroid plexus. The authors proposed that ligation of EVA1 by receptors on T cells reinforces cell-cell junctions and restricts subsequent T cell entry. Thus, the passage of one T cell closes the door to followers. In support of this model, the same lab found that an EVA1 knockout mouse could no longer exclude T cells from the brain or B cells from the CSF [27]. In addition, they showed that treatment of HEK cells overexpressing EVA1 with the activating antibody induced intracellular Ca^+2^ which is important for E-cadherin function at cell-cell junctions [35].

**Fig 13:**
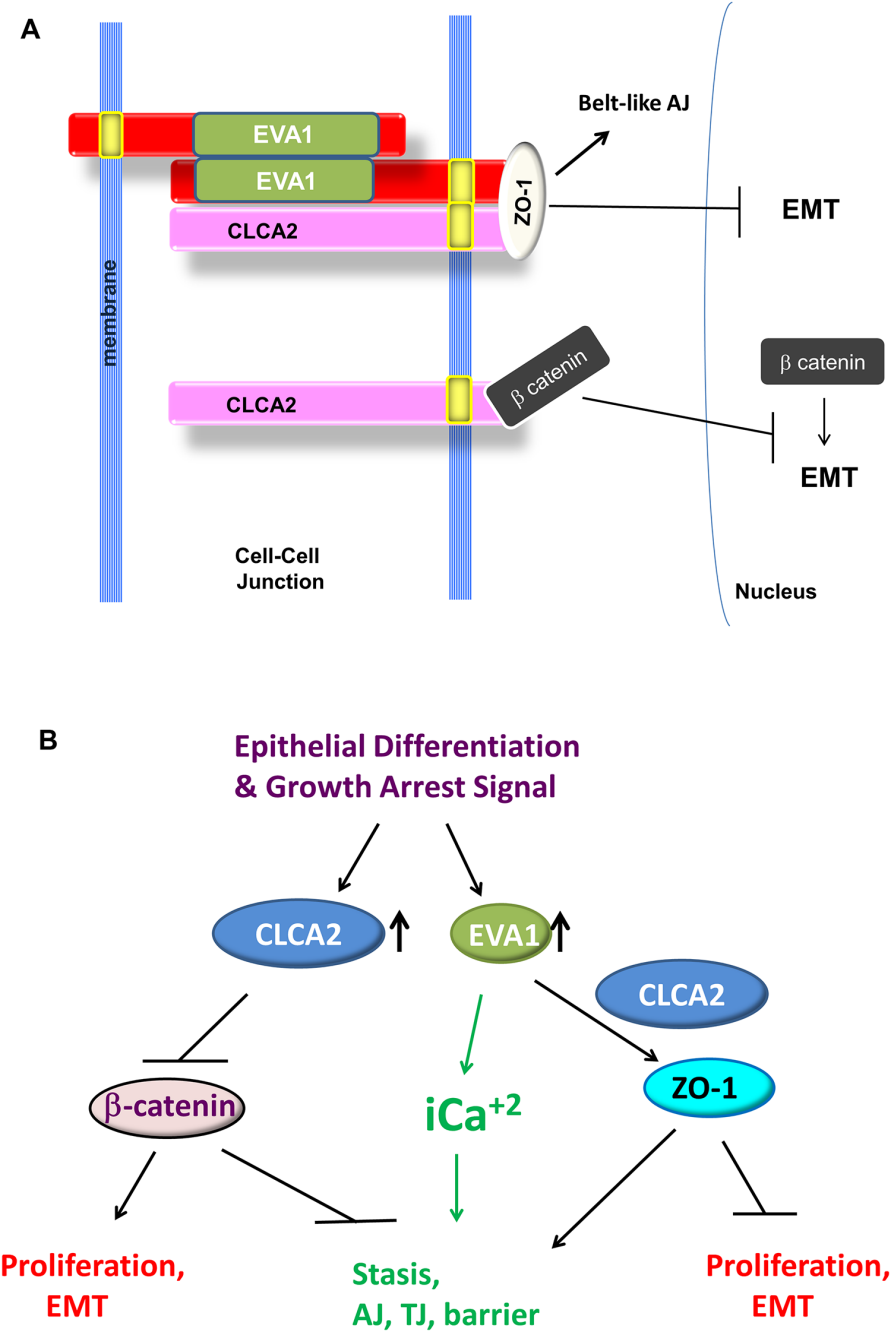
Proposed model for action of CLCA2 and EVA1 at cell-cell junctions. **A**, CLCA2 forms two complexes, one with beta catenin that is independent of EVA1, and the other containing ZO-1 and EVA1, when present. Homophilic ligation of EVA1 may transduce a signal to the cytoskeleton through CLCA2 interaction with ZO-1. CLCA2 is shown as a monomer for simplicity. **B**, pathways by which CLCA2 may promote epithelial differentiation and homeostasis. See text for details.

We found here that, like CLCA2 and E-cadherin, EVA1 is also an epithelial differentiation marker that is required for maintaining differentiation of mammary epithelial cells. EVA1 and CLCA2 both localized throughout the lateral junctions, similar to E-cadherin. To understand how these two junctional proteins mediated differentiation, we screened for TJ and AJ molecule interactions either with CLCA2 or EVA1 or both together. Our results suggest that CLCA2 mediates epithelial differentiation through two different complexes. The first complex contains EVA1 bound to CLCA2 TMS and ZO-1 bound to the cytoplasmic tail of CLCA2 (Fig 13A). In the second complex, beta catenin is bound to the cytoplasmic tail of CLCA2; EVA1 is absent from this complex.

Both complexes have regulatory implications (Fig 13B). ZO-1 is important for tight junction formation as well as formation of belt-like adherens junctions during epithelial differentiation [34]. ZO-1 is a membrane-associated guanylate kinase with multiple protein-protein interaction motifs and an actin binding region [36]. Tsukita found that loss of ZO-1 impaired the activation of Rac1, which is required for belt-like AJ formation during epithelial polarization [34]. These results suggest that the complex of CLCA2 and EVA1 with ZO-1 may promote maturation of AJ and epithelial polarization.

On the other hand, beta catenin is a signaling effector of Wnt and growth factor receptors and is stabilized by the AKT pathway [37]. Its transcriptional targets include effectors of proliferation and mesenchymal transcription factors [38–41]. We found that ectopic expression of CLCA2 destabilizes beta catenin and downregulates these genes. Beta catenin stability is known to be regulated by a cytoplasmic complex containing GSK3, axin, and APC; disruption of this complex is linked to progression of cancers of the colon and other organs [42]. It is possible that CLCA2 cytoplasmic tail participates in this complex, perhaps tethering it to the plasma membrane. These results further suggest that CLCA2 may be useful in damping beta catenin activity in Wnt-dependent cancers.

An interesting teleological question is why cells need CLCA2 to link cell-cell adhesion to beta catenin signaling when E-cadherin already performs this function. E-cadherin appears to be one of the initial interactors when cells meet [43–45]. However, these contacts between membranes are sporadic and do not engage the cytoskeleton [46–48]. Maturation of these contacts into an adhesion belt linked to cytoskeletal actin requires the release of intracellular calcium, which promotes association of ZO-1, AJ, and desmosomes with the cytoskeleton and mobilizes occludin to TJ [49, 50]. It is possible that EVA1-EVA1 contacts occur after E-cadherin initial contacts, and EVA1, with or without CLCA2, is responsible for the burst of intracellular calcium required for maturation of adherens junctions (Fig 13B). CLCA2 also differs in having an enzymatic ectodomain that might inhibit Wnt or other extracellular signaling factors. In this light, in airway epithelial cells, the CLCA1 diffusible ectodomain was shown to enhance the activity of Ano1, activate mucus secretion and transdifferentiation, and activate macrophages [51, 52, 53]. It is tempting to speculate that CLCA2 performs similar functions in mammary epithelium. These possibilities remain to be tested.

The subcellular localization of CLCA2 has never been investigated in HMEC because of a lack of specific antibodies and low protein expression. Here we first used epitope tags to document basolateral localization of ectopic CLCA2 in HEK293 cells and then took advantage of a recently developed antibody to show lateral localization of endogenous CLCA2 in MCF10A. Accordingly, others have reported junctional localization in esophageal squamous epithelium [54] and basal localization in hemidesmosomes of corneal epithelium [55]. This pattern may be significant because basal and lateral adhesion signaling are coordinated through multiple pathways to promote or inhibit cell migration [56], suggesting a possible role for CLCA2 in cross-talk between AJ and hemidesmosomes. In addition to membrane expression, both CLCA2 and EVA1 were present in intracellular vesicles. Thus, we cannot rule out additional, cytoplasmic functions for either protein.

What is the functional significance of the interaction between these proteins? Our finding that the EVA1-CLCA2 interaction occurs between the transmembrane segments suggests that the interaction links the activities of the two proteins. Such allosteric interactions may affect localization, dimerization, or activity of the protein partner [57]. Thus, CLCA2 might promote cell-cell junction formation by localizing EVA1, or EVA1 may direct CLCA2 to the cell-cell junction. Another possibility is that CLCA2 could regulate EVA1 dimerization or ligation to a partner on an opposing cell; or that EVA1 may regulate CLCA2 dimerization and cleavage. Heterodimer formation might prevent CLCA2-CLCA2 interaction and cleavage. Further studies are needed to address these possibilities. Whatever its purpose, the conservation of the CLCA2 TMS primary sequence in mammals suggests that this interaction has played an important role in the evolution of mammalian epithelia.

The EVA1-CLCA2 complex may also play an important role in paracellular trafficking of immune cells. One of the functions of epithelial cell-cell junctions in all organs is to regulate the trafficking of immune cells into the tissues [58]. From the work of Carrithers, EVA1 seems designed to interact with T cells at cell-cell junctions. It is possible that CLCA2 is also involved in EVA1 barrier function at the choroid plexus and that the complex performs a similar barrier or immune filtering function in breast and other tissues. It is tempting to speculate that the metalloprotease activity of CLCA2 may be used by EVA1 to regulate the passage of such cells into tissues in the course of routine immunosurveillance or in response to stress. Thus, in addition to the effects on epithelial differentiation, the loss of EVA1 and/or CLCA2 in breast epithelium might impair immunosurveillance that normally suppresses cancer emergence.

## Conclusion

By surrogate genetics we have identified an important cell-cell junctional signaling mechanism that remained undetected until now because it does not appear to exist in non-mammalian genetic systems. The data fill a gap in our understanding of how junctional signaling suppresses the activity of beta catenin which runs amok in invasive cancers. Future studies to identify other binding partners at cell-cell junctions and functional studies of the role of CLCA2 metalloprotease activity on the organization of lateral junctions may reveal new tumor suppressive pathways that can be exploited in cancer prevention or therapy.
